# Complete Genome Sequence of Foot-and-Mouth Disease Virus Serotype SAT3 Zimbabwe/4/81

**DOI:** 10.1128/genomeA.00801-16

**Published:** 2016-08-25

**Authors:** Su-Hwa You, Hyun Mi Pyo, Seo-Yong Lee, Mi-Kyeong Ko, Joo-Hyung Choi, Sung Ho Shin, Kwang-Nyeong Lee, Byounghan Kim, Jong-Hyeon Park

**Affiliations:** Animal and Plant Quarantine Agency, Gimcheon City, Gyeongsangbuk-do, Republic of Korea

## Abstract

The complete genome sequence of a foot-and-mouth disease (FMD) serotype SAT3 virus ZIM/4/81, which belongs to a topotype 1 (SEZ), is reported here.

## GENOME ANNOUNCEMENT

Foot-and-mouth disease (FMD) is a highly contagious vesicular disease in cloven-hooved ungulates (1, 2). Countries where the FMD outbreaks occur have implemented a culling policy and vaccination to contain the disease (3), but the FMD epidemic prevails in many countries, according to the World Reference Laboratory for Foot-and-Mouth Disease (WRLFMD [http://www.wrlfmd.org/fmd_genotyping/]).

A causative agent of FMD is a single-stranded positive-sense RNA virus that belongs to the genus *Aphthovirus* of the *Picornaviridae* family (4). FMD virus (FMDV) is divided into seven antigenically distinct serotypes, A, O, C, Asia 1, and South African Territories (SATs) 1, 2, and 3.

We took initiative to develop FMD vaccines to counter the disease after the recent FMD outbreaks in South Korea (5). Although serotype C and SAT 1, 2, and 3 FMDVs are at low probability to be introduced in Asian countries, these serotype FMDVs were included as vaccine antigens in an antigen bank for emergency use. As for serotype SAT3 FMDV, Zimbabwe/4/81 (ZIM/4/81) was received from the WRLFMD. Although ZIM/4/81 has been featured in several FMDV research articles as a representative SAT3 topotype 1 virus, its complete genome sequence was not available. The WRLFMD has a record of an SAT3 FMDV outbreak in Zimbabwe in 1981, but the origin of this virus is not clear (B. E. Clarke, unpublished data).

We report here the complete genome sequence of the ZIM/4/81 strain. Viral RNA was extracted from the cell culture supernatant of BHK-21 cell line, and cDNA was synthesized using an oligo(dT) primer with Superscript III reverse transcriptase (Thermo Fisher Scientific). We designed pairs of primers to produce seven overlapping amplicons spanning the entire viral genome based on the sequence of the SAT3 ZIM/6/91 strain (6). Amplified PCR products were subjected to direct sequencing, and the sequencing reads were assembled into a single contig using BioEdit (version 7.2.3).

The complete genome of ZIM/4/81 is 8,124 nucleotides (nt) in length, including a 996-nt 5′ untranslated region (5′ UTR) with 12 nt of a poly(C) tract of unknown length and a 120-nt 3′ UTR with a ≥27-nt poly(A) tail. A single open reading frame of 7,008 nt was predicted to encode a polyprotein of 2,336 amino acids (aa) containing four structural and 10 nonstructural proteins. A putative polyprotein sequence shared 95 to 97% homologies to known SAT 3 FMDV isolates. The most closely related isolate was ZIM/6/91, which shared 92% nucleotide homology of the complete genome.

### Accession number(s).

The complete genome sequence of FMDV SAT3/ZIM/4/81 has been deposited in GenBank under the accession no. KX375417.
